# Protective behavioral strategies and planned drinking relate to high intensity drinking and consequences at the day level

**DOI:** 10.1016/j.addbeh.2025.108591

**Published:** 2025-12-31

**Authors:** Jennifer E. Merrill, Roselyn Peterson, Christian C. Garcia, Lindy K. Howe, Kate B. Carey, Nancy P. Barnett, Kristina M. Jackson, Mary Beth Miller

**Affiliations:** a Center for Alcohol and Addiction Studies, Department of Behavioral and Social Sciences, Brown University, Providence, RI, USA; b Department of Psychology, Bryant University, Smithfield, RI, USA; c Department of Psychology, University of Cincinnati, Cincinnati, OH, USA; d Rutgers Addiction Research Center, Department of Psychiatry, Rutgers Robert Wood Johnson Medical School, Piscataway, NJ, USA; e Department of Psychiatry, University of Missouri, Columbia, MO, USA

**Keywords:** Alcohol Use, Protective Behavioral Strategies, Young adults, Ecological Momentary Assessment

## Abstract

**Background:**

Heavy episodic drinking (HED; 4 + [females]/5 + [males] drinks/occasion), high-intensity drinking (HID; 8 + [females]/10 + [males] drinks/occasion), and drinking events that are planned are all associated with negative consequences. Protective behavioral strategies (PBS) are techniques to minimize alcohol-related consequences. In this day-level study, we hypothesized (1) PBS use would be associated with safer same-day drinking (lower odds of HID and negative consequences), and (2) risks of HID and consequences associated with planned drinking would be reduced on days with higher PBS use. Additionally, (3) on HID days, having planned to engage in HID was hypothesized to relate to use of fewer PBS.

**Method:**

Young adults (*n* = 203, 57 % female) completed a baseline assessment and 28-day ecological momentary assessment of drinking intentions, and number of drinks.

**Results:**

In total, 2,467 drinking days were captured (52% planned, 27% HID). Using more PBS was associated with lower odds of HID (relative to HED but not moderate drinking), and fewer consequences, partially supporting our first hypothesis. PBS did not moderate effects of planned drinking on HID or negative consequence odds. On planned (vs unplanned) HID days, fewer PBS were used, supporting our third hypothesis.

**Conclusion:**

Planning to drink is linked reliably to heavier drinking and negative consequences, but day-level associations between PBS and risky drinking are complex. PBS appear to have less impact on consequences when drinking is planned. When HID in particular is planned, fewer PBS are used. For days when HID is planned, real-time reminders of PBS may add value to intervention efforts.

## 1. Introduction

Alcohol is one of the most commonly used substances in the United States (Nehring et al., 2025). Young adulthood (defined here as age 18–29), is a distinct phase of life characterized by identity formation, instability, and self-focus (Arnett, 2005). This same phase of life is also associated with the highest prevalence of past 12-month alcohol use, high-risk drinking, and alcohol use disorder (Grant et al., 2017). Heavy episodic drinking (HED, 4+/5 + drinks for females/males; Wechsler & Austin, 1998) has been linked to outcomes such as passing out and acting obnoxious or rude (Jackson, 2008). High-intensity drinking (HID; 8+/10 + for females/males; Patrick & Terry-McElrath, 2016 is associated with even worse consequences, including severe injury, overdose, and death (Hingson & White, 2013; Patrick et al., 2016).

## 2. Protective behavioral Strategies: Mitigation of heavy drinking and negative outcomes

Protective behavioral strategies (PBS) are strategies individuals use to minimize the quantity of alcohol consumed and/or the experience of related negative consequences (Martens et al., 2005; Peterson et al., 2021; Treloar et al., 2015). The most commonly used measure of PBS categorizes strategies into three subtypes: (1) *Manner of Drinking* (e.g., drinking slowly, rather than gulping; avoiding shots), (2) *Stopping/Limiting Drinking* (e.g., leaving a party at a predetermined time, establishing a drink limit), and (3) *Serious Harm Reduction* (e.g., only going out with trusted friends or having a designated driver; Martens et al., 2005; Treloar et al., 2015). Overall, individuals who use more PBS report lower levels of drinking and fewer alcohol-related consequences than those who use fewer PBS (Martens et al., 2005; Peterson et al., 2021).

When examining PBS as risk reduction strategies at the day-level, *Manner of Drinking* strategies have been consistently associated with less alcohol consumption (Lewis et al., 2012; Lewis et al., 2015; Pearson et al., 2013). However, effects of other PBS subtype results are mixed and somewhat counterintuitive. Some day-level research suggests use of *Serious Harm Reduction* strategies relate to *heavier* consumption (Lewis et al., 2015; Linden-Carmichael et al., 2019) and/or more negative consequences (Howard et al., 2024; Lewis et al., 2012; Lewis et al., 2015; Pearson et al., 2013). *Stopping/Limiting Drinking* strategies have been linked to *increased* consumption of alcohol, especially during Spring Break days (Lewis et al., 2015), but also to fewer day-level consequences (Braitman et al., 2017; Lewis et al., 2012). Therefore, while PBS should theoretically protect against reaching the heaviest levels of drinking (e.g., high intensity drinking) and reduce risk for consequences, more day-level research is needed to clarify whether, for which outcomes (i.e., consumption vs consequences), and under what conditions (i.e., potential interactions between PBS and other day-level predictors) this is the case. To our knowledge, no studies have looked at the total number of PBS (regardless of subtype) as a predictor of same-day drinking outcomes. Doing so could inform interventions by suggesting that increasing one’s overall repertoire of PBS, in addition to focusing on any specific type, may be valuable.

## 3. Protective behavioral strategies as a potential moderator of planned drinking

Intentions to consume alcohol (i.e., planned vs. unplanned drinking) are a key factor in alcohol-related harm among young adults (Ajzen, 1991). Recent day-level studies have shown planned (vs unplanned) drinking is associated with higher alcohol consumption (Howe & Finn, 2024; Stevens et al., 2022) and more negative outcomes (Lauher et al., 2020; Stevens et al., 2022). In contrast, in the one study to date that has examined intentions to engage in *heavy* drinking (either HED *or* HID), heavy drinking that was *unplanned* was associated with more same-day negative consequences than all other drinking days (e.g., planned heavy drinking, drinking less than intended; Fairlie et al., 2019). As such, examining the nuance of planned and unplanned levels of alcohol use may be relevant for subsequent research aimed at understanding the development of alcohol-related problems. However, limited work has examined this in samples recruited for heavy drinking.

The extent to which planned drinking is risky could be dependent on the use of PBS. Intuitively, the risk posed by planning to drink (or drink heavily) should be mitigated if someone also uses strategies to avoid negative consequences (in other words, the effect of intentions on outcomes may be moderated by use of PBS). In a recent study, all three subtypes of PBS were similarly associated with heavier drinking and negative consequences regardless of whether drinking was planned or unplanned (Howard et al., 2024). However, participants were all college students who did not necessarily engage in HID. Arguably, PBS may be especially important among those who engage in HID – and on days of HID, when use of PBS is less likely (Linden-Carmichael et al., 2019).

## 4. The current study

This study extends previous findings by examining associations among daily protective behavioral strategy use, planned drinking, drinking levels, and negative consequences using multiple day-level reports. We hypothesized that: (H1) more PBS will be associated with lower odds of HID (relative to HED or moderate drinking) and negative consequences, and (H2) PBS will moderate the effect of planned vs unplanned drinking on day-level drinking and consequences. Specifically, we expected planned drinking would be less strongly related to consumption level and consequences when more (vs fewer) PBS were used. On days when participants engaged in HID, we also hypothesized H3) having planned to engage in HID would be associated with fewer PBS. Given nuanced findings regarding the influence of each PBS subtype (e.g., Lewis et al., 2015; Pearson et al., 2013), we do not present hypotheses but explore associations with PBS subtypes.

## 5. Method

### 5.1. Participants

Participants (*n* = 203) were recruited using social media, print advertisements, listservs, and word of mouth. Eligibility required: (1) ages 18 to 29 years, when heavy drinking is most prevalent, (2) 2 + HID days (8+/10 + drinks for females/males) and at least 1 lighter-drinking day in the last 30 days (to observe adequate observations of HID but also variability in level of drinking), (3) smartphone ownership to complete app-delivered self-report surveys, and (4) proximity to the study site (in order to attend an in-person baseline session). To ensure ability to complete a complex study protocol, individuals were excluded if they reported (1) past 6-month treatment for substance use disorder or serious mental illness (e.g., schizophrenia, bipolar disorder), or (2) past 6-month symptoms of a serious mental illness. Of 204 who completed data collection, one did not report any drinking across the 28 days and was excluded from analyses.

## 6. Procedures

All procedures were approved by the university’s Institutional Review Board. Following an online screener, participants were invited to a Zoom session to confirm identification and eligibility and provide informed consent. They then completed a baseline assessment, attended an in-person orientation, and began a 28-day ecological momentary assessment (EMA) protocol. Each day, a morning report was sent at 7am and available until 5 pm. An evening report was sent at 5 pm and available until 11 pm. Participants also were instructed to self-initiate a drinking report upon beginning the first drink of the day. They were then prompted to complete follow-up drinking reports every 45 min during drinking, and once more after indicating they were no longer drinking.

## 7. Baseline measures

### Demographics.

Participants reported sex, gender, age, race, ethnicity, and student status.

## 8. EMA Measures

### Alcohol use.

Morning reports asked, “Did you drink yesterday?” (yes/no). If yes, participants indicated which types of drink(s) they consumed via check-all-that-apply (standard beer/cider, high alcohol-by-volume beer/cider, hard seltzer, wine/champagne, mixed drink, straight liquor, other). They then indicated how many standard drinks consumed of each drink type (e.g., standard drinks of wine/champagne), facilitated by beverage-specific standard drink images. Each drinking day was categorized as moderate drinking (<4/5 drinks for females/males), HED (4–7 drinks for females; 5–8 for males), or HID (8/10 + drinks for females/males).

### Planned vs unplanned drinking.

On both morning and evening reports, if alcohol had not already been used, participants responded to the item, “I intend to drink today,” with options from strongly disagree (0) to strongly agree (4). We used these answers to code any subsequent drinking as planned or unplanned. Responses of 0 through 2 were coded as unplanned drinking (0), and responses of 3 or 4 were coded as planned drinking (1). We primarily used the evening report of intentions to code intentions for subsequent drinking. In the absence of an evening report of intentions (due to missing the report or having already consumed alcohol), we used intentions reported in the (same day) morning report.^2^

### Planned vs unplanned high-intensity drinking.

At the first self-initiated drink report of a day, participants were asked to confirm whether it was truly their first drink. If so, they received the sex-specific item, “I intend to drink 8 (females)/10 (males) or more standard drinks during this drinking episode.” Response options and coding for planned/unplanned HID paralleled those described above. Scoring was applied only to days where HID was reported on the next day morning report (i.e., confirming that HID did occur), allowing a comparison of HID days that were planned vs unplanned.

### Protective behavioral strategies.

At each morning report where prior-day drinking was endorsed, participants received a modified version of the Protective Behavioral Strategy Scale-20 (PBSS-20; Martens et al., 2005; Treloar et al., 2015), with checklists assessing whether they used any of 12 strategies across the three subscales. *Stopping/Limiting Drinking* items included “planned not to exceed set # of drinks,” “stopped drinking at predetermined time,” “drank water,” “alternated with nonalcoholic drinks”, and “asked someone to tell you when you’d drank enough.” *Manner of Drinking* items included “avoided mixing different types of alcohol,” “drank slowly rather than gulping or chugging,” “avoided trying to keep up with or out-drink others,” “didn’t play drinking games when had opportunity” and “didn’t take shots of liquor when offered.” *Serious Harm Reduction* items were “ate just before or while drinking” and “didn’t mix alcohol and drugs when available.” Items were summed (out of a possible total of 12) for total PBS, and each subscale was coded 1 (any) vs 0 (none).

### Negative consequences.

A count of consequences was derived using two sources of data. First, during each real-time drinking report, participants were asked, “Have you had any of these experiences since you started this drinking episode?” with a “check all that apply” list including: embarrassed self, was rude or obnoxious, hurt or injured self by accident, mood worsened, felt too drunk, felt nauseous, vomited, behaved aggressively, other, and “none of these.” Second, on next-day evening reports, if prior-day drinking was endorsed, participants were asked, “During or after drinking yesterday, which of the following experiences did you have?” with a “check all that apply” list containing the same items as in real-time reports, but with additional options for regretted romantic/sexual experience, slept worse, neglected important obligation, hangover, and drove after knowingly having too much to drink to drive safely. Also on evening reports, a separate checklist assessed potential types of alcohol-induced memory loss (block of time I can’t recall at all, memories clear only after reminder, “fuzzy” memories) and no memory loss. Any response other than “no memory loss” was counted as one consequence representing memory loss. These additional consequences were not assessed (and all others reassessed) until next-day evening reports given prior work in young adults who drink heavily demonstrates blackouts (and possibly other consequences) may not be recalled upon first waking (Merrill et al., 2019). A count of different consequences reported – either in real-time or next day – was created for analysis.

## 9. Analytic plan

Hierarchical generalized linear models were conducted in the HLM 7.0 program (Raudenbush et al., 2013), with nesting of days (Level 1) with participants (Level 2). Models predicting day type were conducted on *n* = 2,467 days, models predicting consequences were conducted on *n* = 2,354 days, and models predicting PBS were conducted on a subsample of 144 participants and n = 414 HID days. Type of drinking day (moderate, HED, HID) was tested as a multinomial outcome, with high-intensity drinking as the referent group (HID = 2, moderate = 1, HED = 0). Count variables (negative consequences, total PBS) were tested with a Poisson distribution including an overdispersion parameter.

In models testing H1 (effects of PBS on risky drinking), total PBS was the primary Level 1 predictor. In models testing H2 (interactions), planned (vs unplanned) drinking and a Level 1 × Level 1 interaction between planned drinking and PBS were entered. In models testing H3, planned (vs unplanned) HID was entered as the primary Level 1 predictor of PBS. Level 1 covariates included day in the study (1–28) and weekend (Friday/Saturday = 1, Sunday through Thursday = 0), and in models predicting negative consequences, total drinks consumed. At Level 2, we covaried student status (1 = currently enrolled in 4-year college, 0 = all others). Additionally, aggregated variables corresponding to the primary predictors (e.g., mean PBS, proportion of drinking days that were planned) were modeled at Level 2 to disaggregate day-from person-level effects of these variables.

Across models, continuous predictors were person-centered at Level 1 and grand-mean centered at Level 2.^3^ We interpreted models that relied on robust standard errors in interpretation of effect significance. Intercepts were modeled as random. Random slopes were tested and retained when significant. All models were replicated to explore each PBS subtype. Given this required 18 tests, we applied the Benjamini-Hochberg (BH) procedure for multiple comparisons (Benjamini & Hochberg, 1995). The largest p-value that remained smaller than its BH critical value, and that was therefore considered significant, was p = 0.002.

## 10. Results

### Descriptives.

**Fig. 1.**
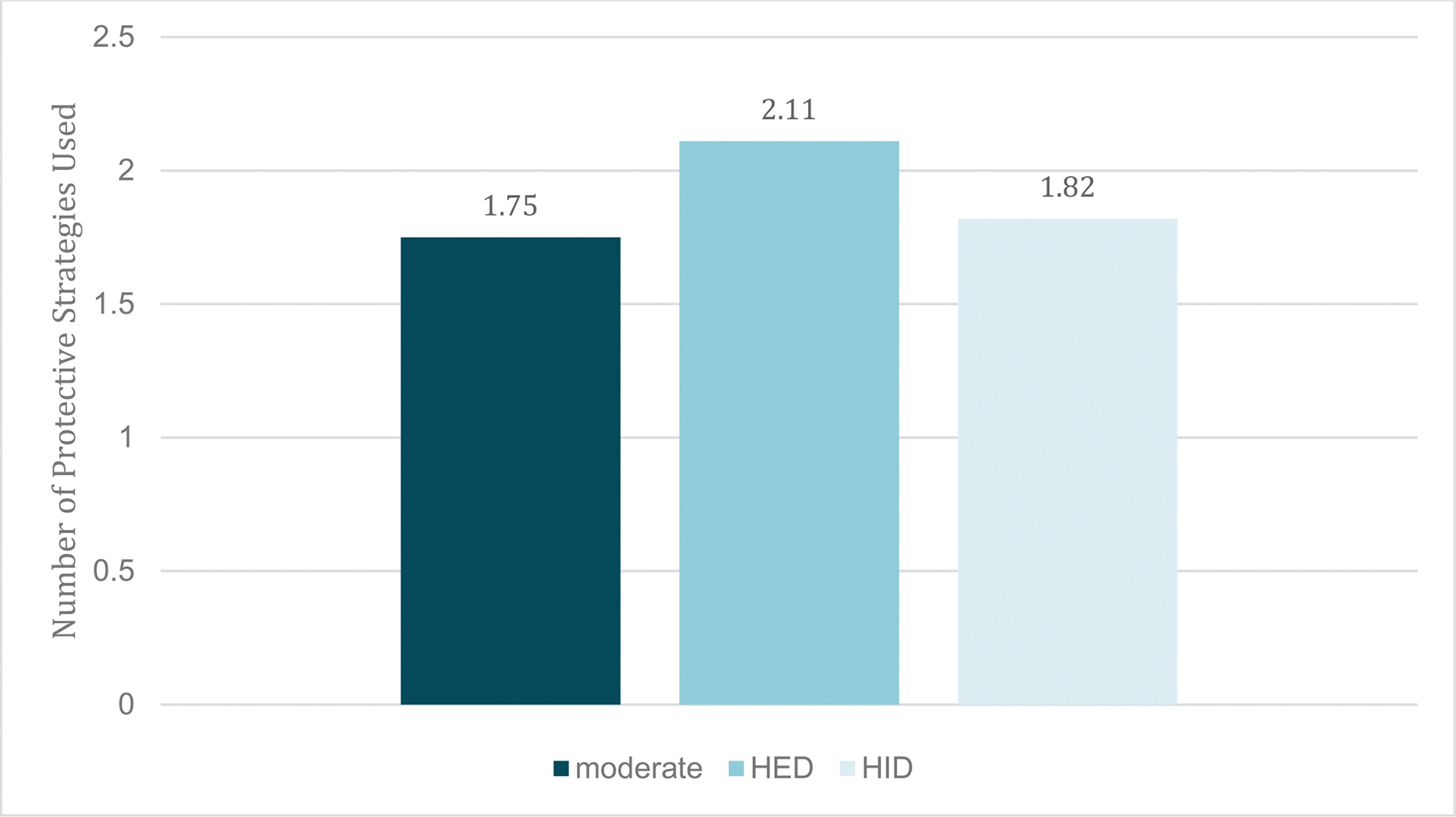
Total Protective Behavioral Strategies by Type of Drinking Day. *Note.* HED = heavy episodic drinking (i.e., 4 + for females and 5 + drinks for males); HID = high intensity drinking (i.e., 8 + for females and 10 + for males).

**Fig. 2.**
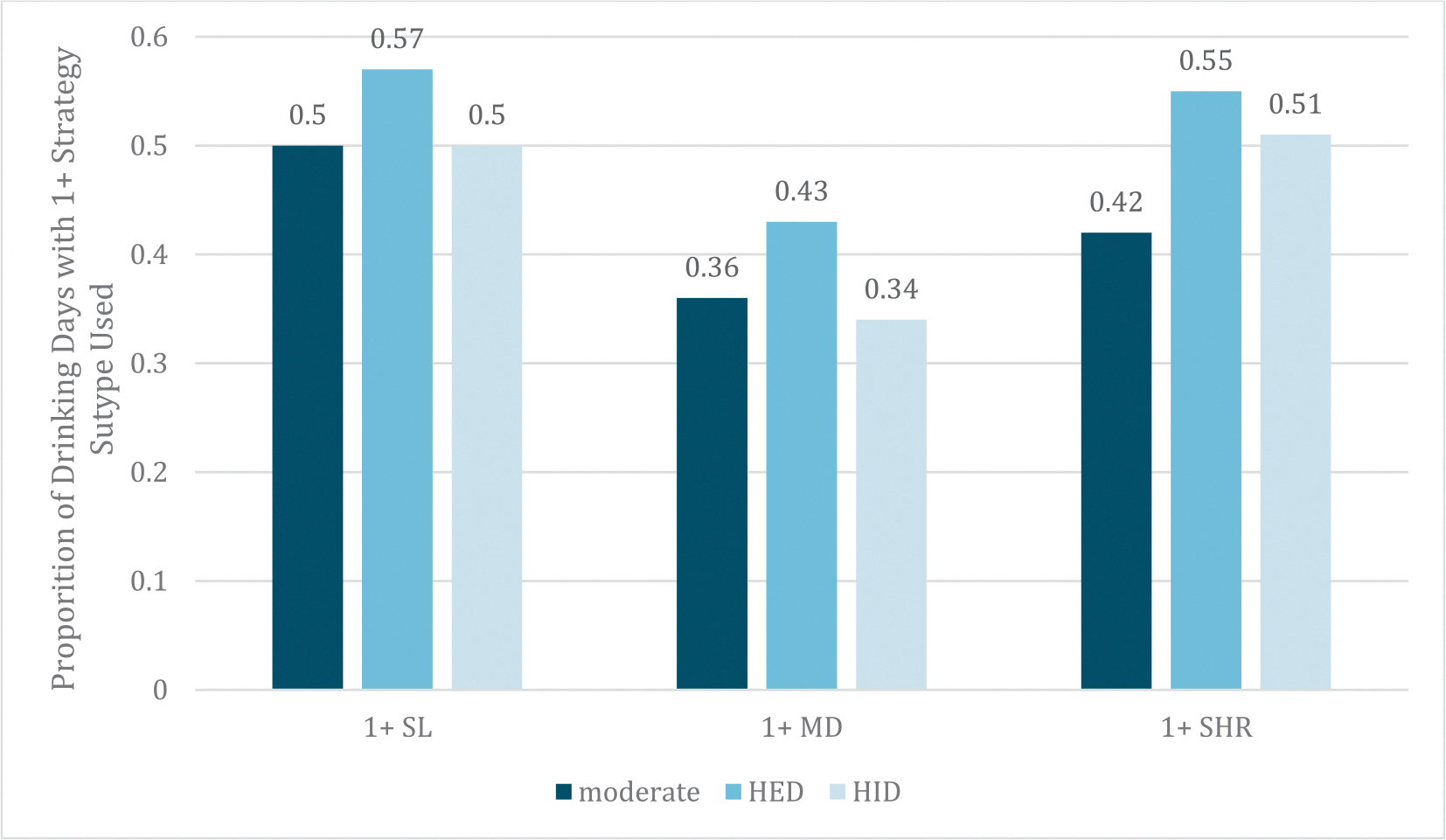
Protective Behavioral Strategy Subtype Use by Type of Drinking Day. *Note.* PBS subtypes are scored as any use (1) vs none (0); as such, numbers represent proportion of days used. SL = stopping/limiting drinking; MD = manner of drinking; SHR = serious harm reduction; HED = heavy episodic drinking (i.e., 4 + for females and 5 + drinks for males); HID = high intensity drinking (i.e., 8 + for females and 10 + for males).

Participants (*n* = 203, 57.1 % female, age *M* = 22.06, *SD* =2.76) self-identified as Asian (7.4 %), Black (5.4 %), Native American/Alaskan (; 0.5 %), White (73.9 %), “other” (3.0 %), multiracial (9.4 %), and Latino/a/x(9.4 %); and half (53.7 %) were enrolled in 4-year college. Across 28 days, participants completed a total of 5439 (95.7 %) morning reports and 5309 (93.4 %) evening reports. Prior-day drinking was reported on 2468 morning reports (45.5 %). Of those, 1927 were accompanied by a prior-day real-time first drinking report (78.1 %). Drinking days averaged 6.28 (*SD* = 4.36) drinks; 51.6 % of drinking days were planned (vs unplanned). In the subset of 144 participants reporting planned or unplanned HID, 36.7 % of all HID days were planned. Rates of PBS use are shown in Figs. 1–2. Intraclass correlations (ICCs) for PBS were: total PBS (0.57), any *Stopping/Limiting Drinking* (0.40), any *Manner of Drinking* (0.40), and any *Serious Harm Reduction* (0.37). In total, 27.0 % of drinking days were HID, 35.0 % were HED, and 39.5 % were accompanied by 1 + negative consequence. Mean consequences per day was 0.93 (*SD* = 1.59, range 0–12). The percentage of days with each type of PBS were: any *Stopping/Limiting Drinking* (52.6 %), any *Manner of Drinking* (38.1 %), and any *Serious Harm Reduction* (49.1 %).

## 11. PBS as predictors

More total PBS use was related to lower odds of HID relative to HED (partially supporting H1) but did not differentiate HID from moderate drinking (Table 1). As hypothesized, total PBS was also associated with fewer negative consequences. For the PBS subscales, use of at least one *Serious Harm Reduction* strategy was associated with lower odds of moderate drinking vs HID (Supplementary Table 1) but did not differentiate HED and HID and was unrelated to consequences. Use of at least one *Stopping/Limiting* strategy was associated with lower risk of consequences (Supplementary Table 2). Use of any *Manner of Drinking* strategy were unrelated to drinking categories or consequences (Supplementary Table 3).

## 12. PBS as Moderators of planned drinking

Contrary to H2, total PBS did not moderate effects of planned drinking on heavier drinking or consequence odds (Table 2). The conditional effect of PBS indicated that at one’s average levels of PBS, planned drinking increased the odds of HID relative to both HED and moderate drinking, and was related to higher consequence odds. Using the BH-corrected p-values, no PBS subtypes moderated the effect of planned drinking on heavier drinking or consequence odds (Supplementary Tables 4–6).

Supplementary examination of planned drinking on protective behavioral strategy use. Given interactions between PBS and planning were largely unsupported, we conducted a supplementary examination of how planning may relate to use of PBS. Planned drinking was unrelated to total PBS use (*Event Rate Ratio [ERR]* = 1.00, 95 % CI = 0.93–1.06, *p*=.920). When the PBS subscales were considered, planned drinking also was unrelated to any *Stopping/Limiting Drinking* (*OR* = 0.90, 95 % CI = 0.72–1.14, BH adjusted p = 0.536), *Manner of Drinking* strategy (*OR* = 1.14, 95 % CI = 0.91–1.42, BH adjusted p = 0.424), or *Serious Harm Reduction* strategy (*OR* = 1.32, 95 % CI = 1.05–1.66, BH adjusted p = 0.102*).*

## 13. Planning to engage in HID and PBS

**Fig. 3.**
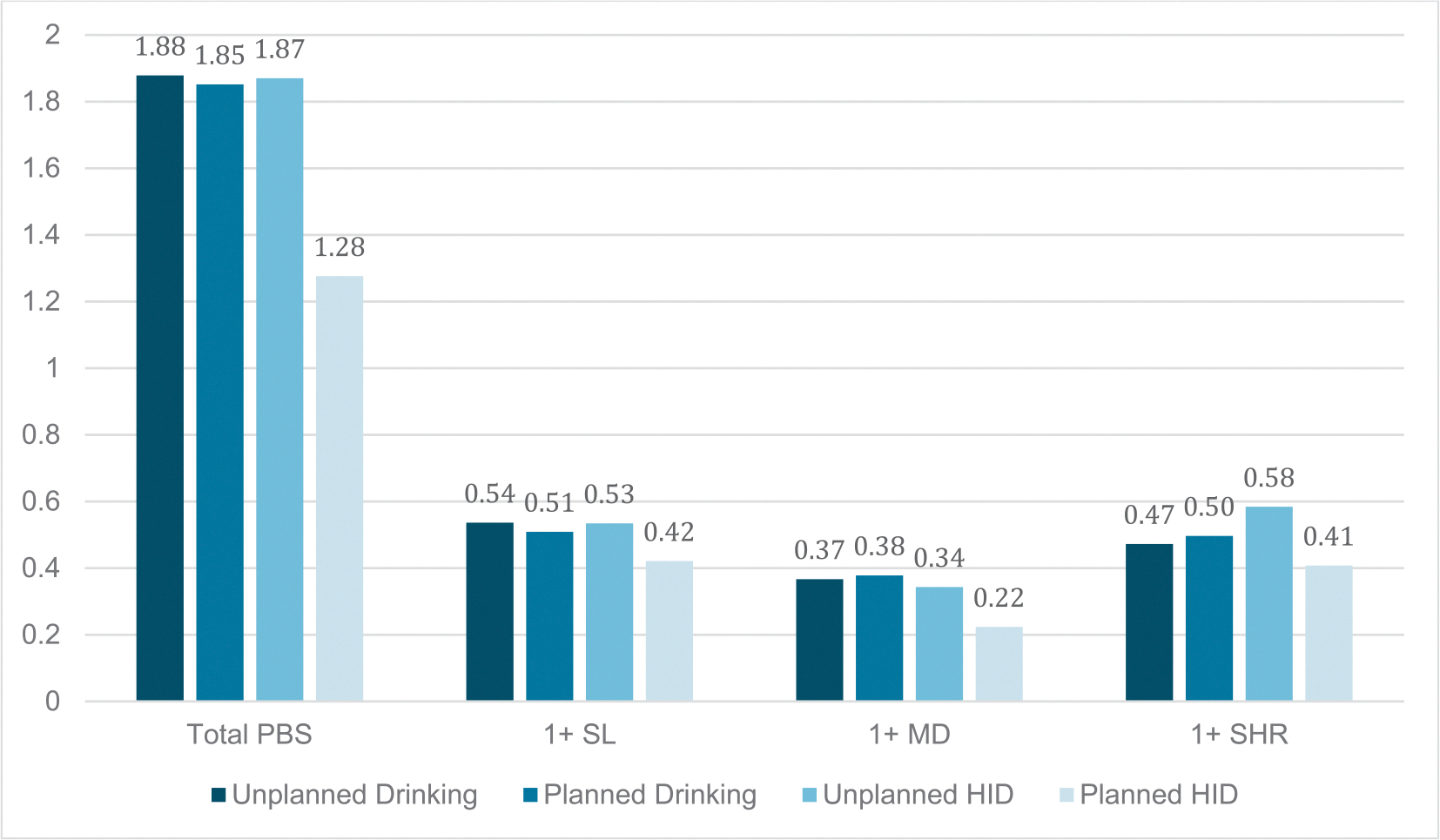
Total Protective Behavioral Strategy Use on Unplanned vs Planned Drinking Days and Unplanned vs Planned High-Intensity Drinking Days. *Note.* Total PBS is the sum of PBS of any type used per drinking day. PBS subtypes are scored as any use (1) vs none (0); as such, numbers represent proportion of days used. HID = high intensity drinking (i.e., 8 + for females and 10 + for males), SL = stopping/limiting drinking; MD = manner of drinking; SHR = serious harm reduction. HID are from a subsample of participants.

In support of H3, planned (vs unplanned) HID was related to use of fewer total PBS (*ERR* = 0.83, 95 % CI = 0.69–0.99, *p* = 0.042). Regarding PBS subscales, planned HID was unrelated to odds of use of any *Stopping/Limiting Drinking* (*OR* = *0.82,* 95 % CI = 0.50–1.34, BH adjusted p = 0.378), *Manner of Drinking* (*OR* = 0.65, 95 % CI = 0.37–1.15, BH adjusted p = 0.355), or *Serious Harm Reduction* strategies (*OR* = 0.66, 95 % CI = 0.35–1.24, BH adjusted p = 0.545). Fig. 3 shows use of PBS on days with planned versus unplanned drinking (any) and HID.

## 14. Discussion

This study examined effects of day-level PBS use, planned drinking, and their interactions on alcohol use and negative consequences. Planned drinking elevated the risk of heavier drinking and negative consequences, and PBS lowered the likelihood of HID and consequences. Results also suggested that using PBS does not help to lessen the impact of planned drinking on heavier use and negative consequences, but that young adults may use fewer PBS when planning to engage in very heavy drinking.

## 15. Effects of PBS on drinking Behavior

Our hypothesis that more PBS use would be associated with lower odds of HID relative to HED, and with fewer consequences, was supported. However, PBS did not differentiate odds of HID versus moderate drinking. This non-linear pattern indicates that the highest level of PBS use occurred on HED, not HID, days. One interpretation is that the use of harm reduction strategies protects against reaching HID levels. However, an alternative explanation is that, when individuals engage in HID, they are too intoxicated, too unaware, or simply unmotivated to implement PBS effectively. Indeed, previous work notes that individuals may not always employ such harm-reduction strategies, especially at higher levels of consumption (Linden-Carmichael et al., 2019). Specifically, some research suggests PBS are particularly useful in relatively *light* drinkers (Li et al., 2020) and our research extends this finding to day-level data.

A different pattern was observed for *Serious Harm Reduction* PBS. Use of at least one such strategy was related to higher odds of HID compared to moderate drinking. However, use of strategies in this subscale did not significantly differentiate between HED and HID, consistent with prior day-level work (Linden-Carmichael et al., 2019). Strategies like not mixing alcohol and drugs or eating just before/while drinking, are most relevant at the highest levels of consumption. Thus, it is promising that young adults choose to use PBS when engaging in heavy drinking (HED or HID). Still, our data do not suggest a day-level link between *Serious Harm Reduction* PBS and negative consequences.

The *Stopping/Limiting Drinking* and *Manner of Drinking* PBS subtypes did not differentiate the type of drinking day. In prior work, these two PBS subtypes were also unrelated to drinking quantities and consequences and remained consistent regardless of plans to drink (Howard et al., 2024). Findings from the present study fill a gap in the literature by clarifying a role for considering the total number of PBS employed. Although individual PBS subtypes may show limited utility in distinguishing drinking patterns, examining total PBS provides a clearer picture of how PBS can protect against HID and negative consequences at the day level.

## 16. PBS as a moderator of planned drinking

We expected that the effect of planned drinking on drinking and consequence outcomes would *depend on* (i.e., be moderated by) the level of PBS use, and specifically that planned drinking would show lower alcohol consumption and consequences when greater (vs. lower) PBS were used, but this hypotheses was not supported; total PBS use (as well as each subscale) did not moderate effects of planned drinking on either drinking or consequences. Some research indicates that PBS may not negate the effects of intentional heavy drinking in young adults (Howard et al, 2024). It may be that, when someone plans to drink, they lose the ability to implement PBS *effectively*, or that the PBS used may not be sufficient to curb risk. Our other findings also suggest that when planning to drink especially heavily (i.e., HID), young adults may not also plan to engage in protective behaviors. Other contextual and individual level factors, such as one’s drinking environment, social norms, beliefs about alcohol use, or drinking intensity, may also be relevant for PBS’ success in reducing negative consequences (Bravo et al., 2017; Howard et al., 2024; Howe & Finn, 2024; Li et al., 2020). Future work should examine the role such factors have on PBS use effectiveness during planned drinking events.

## 17. Planned drinking, planned high-intensity drinking, and PBS

The total number of PBS used on planned (vs unplanned) HID days was significantly lower, consistent with our hypothesis. PBS are typically employed when individuals aim to decrease alcohol-related harms, consumption, or intoxication. On planned HID days, there may be a *desire* to reach high levels of intoxication. In such cases, one may intentionally *not* eat before drinking and *not* set a drinking limit to ensure HID leads to intoxication. Notably, we did not find evidence that any PBS subtype was more or less likely to be used on planned versus unplanned HID days. It is important to note that models testing planned vs unplanned HID were limited to fewer participants and far fewer days, reducing power.

In post hoc analyses, we examined the effect of *any* planned vs unplanned drinking (i.e., including all drinking amounts) on PBS. Planned drinking (vs. unplanned) was unrelated to total PBS use as well as each subscale. This suggests that while planning to drink does not lead to increased or decreased use of total PBS relative to days when drinking is unplanned. Together, our findings also suggest that the relationship between planning and PBS may be specific to HID, as planned HID was related to use of fewer PBS. Future research examining planned levels of *intoxication* might further clarify this relationship.

## 18. Person-level effects

Hypotheses centered on day-level effects; however, multilevel models also allow examination of person-level effects. The number of PBS a participant used *on average* was unrelated to HID or consequences. Likewise, the proportion of drinking days that were planned was unrelated to these outcomes. Whether PBS are used and whether drinking is planned *on a given day* was more relevant to the prediction of risky drinking in this sample than the extent to which PBS and planning differed between people, across all days measured.

## 19. Limitations and future directions

This study has limitations that bear consideration. First and foremost, as PBS were not assessed in real-time, we cannot establish temporality of associations (e.g., when PBS were used in relation to crossing HED or HID thresholds). The sample of young adults endorsing HID may limit generalizability to lighter drinkers or those with different drinking patterns. As aforementioned, future work should explore additional contextual factors (e.g., peer pressure, location) and drinking motives, which may interact with PBS use and effectiveness. Further, we did not assess intentions to engage in HED and could therefore only compare PBS use on planned versus unplanned HID days (and not planned versus unplanned HED days). Similarly, it is possible that participants reported on intentions to drink after the onset of drinking for a small number of drinking days; however, participants confirmed they had not begun drinking for > 80 % of these reports. We examined only a subset of possible negative consequences of drinking in this study; how PBS and planned drinking influence other outcomes that may take more to develop (e.g., tolerance, interpersonal conflict) cannot be determined here. Additionally, while participants were asked whether they experienced consequences “during or after drinking,” they were not asked to confirm that those experiences were attributable to alcohol. However, prior research shows similar consequences may occur even in the absence of drinking (Emery et al., 2025; Flori et al., 2025).

## 20. Clinical implications

Results have implications for harm-reduction strategies and intervention development among young adults who drink heavily. Given PBS use was associated with a higher odds of HED than HID, interventions could focus on strategies to enhance the effectiveness of PBS among high-intensity consumers, with a focus on expanding one’s full repertoire of PBS and not just a specific subtype. Also, given the risk associated with planned drinking, harm-reduction messaging should emphasize strategies to prevent excessive consumption even when drinking is premeditated.

## Supplementary Material

1

## Figures and Tables

**Table 1:** Effects of total PBS on odds of high-intensity drinking and negative consequences.

	HED vs HID			Moderate vs HID			# Negative consequences		
	OR	95 % CI	*p*	OR	95 % CI	*P*	ERR	95 % CI	*p*

Intercept	2.46	(1.74,3.47)	0<.001	3.25	(2.14,4.94)	0<.001	0.49	(0.39,0.61)	0<.001
Day-level									
Total PBS	**1.11**	**(1.01,1.22)**	**0.033**	0.95	(0.84,1.07)	0.371	**0.94**	**(0.88,1.00)**	**0.049**
Study Day	1.99	(0.98,1.01)	0.591	0.99	(0.97,1.01)	0.250	0.98	(0.97,0.99)	0<.001
Weekend	0.47	(0.37,0.60)	0<.001	0.21	(0.16,0.27)	0<.001	1.20	(1.05,1.37)	0.008
Total Drinks	– -	– -	– -	– -	– -	– -	1.24	(1.21,1.27)	0<.001
Person-level									
Student	1.15	(0.81,1.62)	0.432	1.12	(0.73,1.74)	0.600	1.62	(1.29,2.04)	0.010
Average PBS	1.07	(0.94,1.22)	0.291	0.98	(0.84,1.15)	0.790	1.07	(0.99,1.15)	0.092

*Note.* Significant effects of interest are bolded. In all models, HID is the referent event (coded 2). PBS = protective behavioral strategies; HID = high intensity drinking (i.e., 8 + for females and 10 + for males); HED = heavy episodic drinking (i.e., 4–7 for females and 5–9 drinks for males); OR = odds ratio; CI = confidence interval. Weekend coded 1 or Friday/Saturday and 0 for all other days. Student coded 1 for current 4-year college attendance and 0 for all other. Random effects in the model predicting day type were non-significant and fixed. In the prediction of consequences, all random effects were significant and retained. When planned drinking was added as a covariate at both the day and person-level, effects of total PBS were similar in magnitude and significance.

**Table 2:** Testing total PBS as a moderator of effects of planned drinking on odds of high-intensity drinking and negative consequences.

	HED vs HID			Moderate vs HID			# Negative consequences		
	OR	95 %CI	*p*	OR	95 %CI	*p*	ERR	95 %CI	*p*

Intercept	3.50	(2.29,5.34)	0<.001	7.75	(4.76,12.62)	0<.001	0.48	(0.38,0.62)	0<.001
Day-level									
Total PBS	1.09	(0.95,1.25)	0.240	0.93	(0.79,1.10)	0.419	0.94	(0.84,1.10)	0.241
Planned	**0.52**	**(0.39,0.70)**	**0<.001**	**0.14**	**(0.10,0.20)**	**0<.001**	**1.38**	**(1.17,1.63)**	**0<.001**
Planned × PBS	1.04	(0.87,1.25)	0.660	1.03	(0.81,1.31)	0.792	0.98	(0.82,1.17)	0.621
Study Day	0.99	(0.98,1.01)	0.487	0.99	(0.97,1.01)	0.217	0.98	(0.97,0.99)	0<.001
Weekend	0.52	(0.41,0.67)	0<.001	0.26	(0.20,0.35)	0<.001	1.03	(0.91,1.17)	0.628
Total Drinks	– -	– -	– -	– -	– -	– -	1.22	(1.19,1.25)	0<.001
Person-level									
Student	1.22	(0.86,1.73)	0.275	1.18	(0.74,1.89)	0.494	1.50	(1.18,1.90)	0.001
Prop. Planned Days	0.67	(0.30,1.53)	0.343	0.84	(0.29,2.45)	0.743	0.82	(0.48,1.39)	0.451
Average PBS	1.05	(0.93,1.20)	0.421	0.95	(0.81,1.12)	0.557	1.06	(0.98,1.15)	0.147

*Note.* Significant effects of interest are bolded. In all models, HID is the referent event (coded 2). PBS = protective behavioral strategies; HID = high intensity drinking (i.e., 8 + for females and 10 + for males); HED = heavy episodic drinking (i.e., 4 + for females and 5 + drinks for males); OR = odds ratio; CI = confidence interval. Weekend coded 1 or Friday/ Saturday and 0 for all other days. Student is coded 1 for current 4-year college attendance and 0 for all other. Random effects in the model predicting day type were non-significant and fixed. In the prediction of consequences, random effects that were significant (Study Day, Total Drinks) were retained. The effect of planned drinking is specific to one’s average day-level use of PBS. When the interaction is removed from the model, effects of planned drinking were similar: on HED vs HID OR = 0.52 (0.39,0.70); on moderate vs HID OR = 0.14 (0.10,0.19); on consequences ERR = 2.58 (2.15,3.09); all ps < 0.001.

## Data Availability

Data will be made available on request.

## Footnotes

2 Average completion time for the morning report was 10:28am. In total 77% of morning reports were submitted before noon, and 89% before 2pm. On average, participants completed their morning report within 1.5 h of waking.

3 For binary predictors (e.g., planned HID, use of a PBS subtype), 0 (no) vs 1 (yes) coding was retained at Level 1. At Level 2, the proportion of days that were coded 1 was modeled and grand-mean centered.

## Appendix A. Supplementary data

Supplementary data to this article can be found online at https://doi.org/10.1016/j.addbeh.2025.108591.
