# Supplementary material for: Protective behavioral strategies and planned drinking relate to high intensity drinking and consequences at the day level
Source: Addict Behav. Author manuscript; Available in PMC 2026 Feb 7. (PMC12881928; doi:10.1016/j.addbeh.2025.108591)
Supplement: 1 [file NIHMS2135768-supplement-1.docx]

| **Supplementary Table 1** | | | | | | | | | |
| --- | --- | --- | --- | --- | --- | --- | --- | --- | --- |
| *Effects of Any Stopping/Limiting Drinking (SLD) Strategy on Odds of High-Intensity Drinking and Negative Consequences* | | | | | | | | | |
|  | HED vs HID | | | Moderate vs HID | | | # Negative Consequences | | |
|  | OR | 95% CI | *p* | OR | 95% CI | *p* | ERR | 95% CI | *p* |
| Intercept | 2.27 | 1.55, 3.32 | <0.001 | 3.30 | 2.04, 5.33 | <0.001 | 0.58 | 0.46, 0.74 | <0.001 |
| Day-level |  |  |  |  |  |  |  |  |  |
| Any SLD* | 1.22 | 0.91, 1.62 | 0.378 | 0.94 | 0.66, 1.35 | 0.741 | **0.77** | **0.65, 0.91** | **0.018** |
| Study Day | 0.99 | 0.98, 1.01 | 0.348 | 0.99 | 0.98, 1.01 | 0.307 | 0.98 | 0.97, 0.99 | <0.001 |
| Weekend | 0.47 | 0.37, 0.60 | <0.001 | 0.20 | 0.16, 0.27 | <0.001 | 1.18 | 1.04, 1.35 | 0.013 |
| Total Drinks | --- | --- | --- | --- | --- | --- | 1.23 | 1.21, 1.26 | <0.001 |
| Person-level |  |  |  |  |  |  |  |  |  |
| Student | 1.17 | 0.83, 1.66 | 0.360 | 1.13 | 0.73, 1.75 | 0.577 | 1.64 | 1.29, 2.08 | <0.001 |
| Proportion SLD | 1.31 | 0.68, 2.56 | 0.419 | 1.04 | 0.47, 2.29 | 0.926 | 1.91 | 1.26, 2.89 | 0.002 |
| *Note.* Significant effect of interest bolded. In all models, HID is the referent event (coded 2). SLD= Stopping/Limiting protective behavioral strategy; HID=high intensity drinking (i.e., 8+ for females and 10+ for males); HED=heavy episodic drinking (i.e., 4-7 for females and 5-9 drinks for males); OR=odds ratio; CI=confidence interval, ERR=event rate ratio. Weekend coded 1 or Friday/Saturday and 0 for all other days. Student coded 1 for current 4-year college attendance and 0 for all other. Random effects in the model predicting day type were non-significant and fixed. In the prediction of consequences, significant random effects (Study Day, Weekend, Total Drinks) were significant and retained; *Effect of interest; p-values adjusted following Benjamini-Hochberg procedure. | | | | | | | | | |

| **Supplementary Table 2** | | | | | | | | | |
| --- | --- | --- | --- | --- | --- | --- | --- | --- | --- |
| *Effects of Any Manner of Drinking (MD) Strategy on Odds of High-Intensity Drinking and Negative Consequences* | | | | | | | | | |
|  | HED vs HID | | | Moderate vs HID | | | # Negative Consequences | | |
|  | OR | 95% CI | *p* | OR | 95% CI | *p* | ERR | 95% CI | *p* |
| Intercept | 2.21 | 1.53, 3.20 | <0.001 | 3.07 | 1.94, 4.84 | <0.001 | 0.60 | 0.47, 0.77 | <0.001 |
| Day-level |  |  |  |  |  |  |  |  |  |
| Any MD* | 1.41 | 1.02, 1.93 | 0.158 | 1.08 | 0.75, 1.55 | 0.689 | 0.84 | 0.69, 1.02 | 0.240 |
| Study Day | 0.99 | 0.98, 1.01 | 0.479 | 0.99 | 0.98, 1.01 | 0.388 | 0.98 | 0.97, 0.99 | <0.001 |
| Weekend | 0.47 | 0.37, 0.60 | <0.001 | 0.20 | 0.16, 0.27 | <0.001 | 1.09 | 0.96, 1.24 | 0.168 |
| Total Drinks | --- | --- | --- | --- | --- | --- | 1.23 | 1.21, 1.26 | <0.001 |
| Person-level |  |  |  |  |  |  |  |  |  |
| Student | 1.13 | 0.80, 1.60 | 0.477 | 1.14 | 0.73, 1.77 | 0.561 | 1.52 | 1.20, 1.92 | <0.001 |
| Proportion MD | 1.18 | 0.61, 2.31 | 0.616 | 1.15 | 0.50, 2.62 | 0.741 | 1.59 | 1.02, 2.48 | 0.042 |
| *Note.* In all models, HID is the referent event (coded 2). MD= Manner of Drinking protective behavioral strategy; HID=high intensity drinking (i.e., 8+ for females and 10+ for males); HED=heavy episodic drinking (i.e., 4-7 for females and 5-9 drinks for males); OR=odds ratio; CI=confidence interval, ERR=event rate ratio. Weekend coded 1 or Friday/Saturday and 0 for all other days. Student coded 1 for current 4-year college attendance and 0 for all other. Random effects in the model predicting day type were non-significant and fixed. In the prediction of consequences, significant random effects (Study Day, Total Drinks) were significant and retained; *Effect of interest; p-values adjusted following Benjamini-Hochberg procedure. | | | | | | | | | |

| **Supplementary Table 3** | | | | | | | | | |
| --- | --- | --- | --- | --- | --- | --- | --- | --- | --- |
| *Effects of Any Serious Harm Reduction (SHR) Strategy on Odds of High-Intensity Drinking and Negative Consequences* | | | | | | | | | |
|  | HED vs HID | | | Moderate vs HID | | | # Negative Consequences | | |
|  | OR | 95% CI | *p* | OR | 95% CI | *p* | ERR | 95% CI | *p* |
| Intercept | 2.68 | 1.81, 3.96 | <0.001 | 4.76 | 3.00, 7.58 | <0.001 | 0.52 | 0.41, 0.67 | <0.001 |
| Day-level |  |  |  |  |  |  |  |  |  |
| Any SHR* | 0.96 | 0.71, 1.29 | 0.770 | **0.50** | **0.35, 0.71** | **0.018** | 0.91 | 0.77, 1.09 | 0.450 |
| Study Day | 0.99 | 0.98, 1.01 | 0.255 | 0.99 | 0.97, 1.00 | 0.107 | 0.98 | 0.98, 0.99 | <0.001 |
| Weekend | 0.47 | 0.37, 0.60 | <0.001 | 0.21 | 0.16, 0.27 | <0.001 | 1.17 | 1.03, 1.33 | 0.020 |
| Total Drinks | --- | --- | --- | --- | --- | --- | 1.24 | 1.21, 1.27 | <0.001 |
| Person-level |  |  |  |  |  |  |  |  |  |
| Student | 1.14 | 0.81, 1.62 | 0.449 | 1.13 | 0.72, 1.76 | 0.600 | 1.60 | 1.27, 2.03 | <0.001 |
| Proportion SHR | 1.34 | 0.72, 2.51 | 0.358 | 1.86 | 0.81, 4.27 | 0.140 | 1.53 | 1.02, 2.28 | 0.040 |
| *Note.* Significant effect of interest bolded. In all models, HID is the referent event (coded 2). SHR= Serious harm reduction protective behavioral strategy; HID=high intensity drinking (i.e., 8+ for females and 10+ for males); HED=heavy episodic drinking (i.e., 4-7 for females and 5-9 drinks for males); OR=odds ratio; CI=confidence interval, ERR=event rate ratio. Weekend coded 1 or Friday/Saturday and 0 for all other days. Student coded 1 for current 4-year college attendance and 0 for all other. Random effects in the model predicting day type were non-significant and fixed. In the prediction of consequences, all random effects were significant and retained; *Effect of interest; p-values adjusted following Benjamini-Hochberg procedure. | | | | | | | | | |

| **Supplementary Table 4** |  | | |  | | |  | | |
| --- | --- | --- | --- | --- | --- | --- | --- | --- | --- |
| *Testing Any Stopping/Limiting Drinking (SLD) Strategy as a Moderator of Effects of Planned Drinking on Odds of High-Intensity Drinking* *and Negative Consequences* | | | | | | | | | |
|  | HED vs HID | | | Moderate vs HID | | | # Negative Consequence | | |
|  | OR | 95%CI | *p* | OR | 95%CI | *p* | ERR | 95%CI | *p* |
| Intercept | 3.59 | 2.36, 5.46 | <0.001 | 7.80 | 4.81, 12.65 | <0.001 | 0.36 | 0.29, 0.46 | <0.001 |
| Day-level |  |  |  |  |  |  |  |  |  |
| Any SLD | 1.14 | 0.74, 1.74 | 0.549 | 0.70 | 0.40, 1.22 | 0.210 | 1.01 | 0.75, 1.36 | 0.965 |
| Planned | 0.52 | 0.39, 0.70 | <0.001 | 0.14 | 0.10, 0.20 | <0.001 | 1.66 | 1.40, 1.96 | <0.001 |
| Planned x SLD* | 1.03 | 0.59, 1.83 | 0.908 | 1.61 | 0.82, 3.17 | 0.378 | 0.70 | 0.50, 0.99 | 0.166 |
| Study Day | 0.99 | 0.98, 1.01 | 0.243 | 0.99 | 0.97, 1.01 | 0.210 | 0.99 | 0.98, 1.00 | 0.011 |
| Weekend | 0.52 | 0.41, 0.67 | <0.001 | 0.26 | 0.20, 0.35 | <0.001 | 1.06 | 0.94, 1.20 | 0.325 |
| Total Drinks | --- | --- | --- | --- | --- | --- | 1.21 | 1.17, 1.24 | <0.001 |
| Person-level |  |  |  |  |  |  |  |  |  |
| Student | 1.24 | 0.87, 1.77 | 0.226 | 1.18 | 0.75, 1.88 | 0.471 | 0.36 | 0.29, 0.46 | <0.001 |
| Prop. Planned Days | 0.68 | 0.30, 1.52 | 0.341 | 0.85 | 0.29, 2.49 | 0.772 | 0.70 | 0.42, 1.16 | 0.162 |
| Proportion SLD | 1.50 | 0.84, 2.66 | 0.168 | 0.86 | 0.41, 1.80 | 0.690 | 1.33 | 0.93, 1.90 | 0.113 |
| *Note.* In all models, HID is the referent event (coded 2). SLD= Stopping/Limiting protective behavioral strategy; HID=high intensity drinking (i.e., 8+ for females and 10+ for males); HED=heavy episodic drinking (i.e., 4+ for females and 5+ drinks for males); OR=odds ratio; CI=confidence interval, ERR=event rate ratio. Weekend coded 1 or Friday/Saturday and 0 for all other days. Student coded 1 for current 4-year college attendance and 0 for all other. Random effects in the model predicting day type were non-significant and fixed. In the prediction of consequences, random effects that were significant (Study Day, Total Drinks) were retained. The effect of planned drinking is specific to days when one was at their own average proportion of days using SLD; *Effect of interest; p-values adjusted following Benjamini-Hochberg procedure. | | | | | | | | | |

| **Supplementary Table 5** |  | | |  | | |  | | |
| --- | --- | --- | --- | --- | --- | --- | --- | --- | --- |
| *Testing Any Manner of Drinking (MD) Strategy as a Moderator of Effects of Planned Drinking on Odds of High-Intensity Drinking* *and Negative Consequences* | | | | | | | | | |
|  | HED vs HID | | | Moderate vs HID | | | # Negative Consequence | | |
|  | OR | 95%CI | *p* | OR | 95%CI | *p* | ERR | 95%CI | *p* |
| Intercept | 3.61 | 2.36, 5.50 | <0.001 | 7.56 | 4.65, 12.29 | <0.001 | 0.43 | 0.34, 0.55 | <0.001 |
| Day-level |  |  |  |  |  |  |  |  |  |
| Any MD | 1.30 | 0.75, 2.23 | 0.350 | 1.15 | 0.65, 2.03 | 0.638 | 1.0 | 0.74, 1.34 | 0.975 |
| Planned | 0.52 | 0.39, 0.69 | <0.001 | 0.14 | 0.10, 0.19 | <0.001 | 1.47 | 1.25, 1.73 | <0.001 |
| Planned x MD* | 1.17 | 0.61, 2.23 | 0.681 | 0.91 | 0.46, 1.80 | 0.781 | 0.89 | 0.62, 1.28 | 0.628 |
| Study Day | 0.99 | 0.98, 1.01 | 0.386 | 0.99 | 0.97, 1.01 | 0.332 | 0.98 | 0.98, 0.99 | <0.001 |
| Weekend | 0.52 | 0.41, 0.67 | <0.001 | 0.26 | 0.20, 0.35 | <0.001 | 1.0 | 0.99, 1.13 | 0.981 |
| Total Drinks | --- | --- | --- | --- | --- | --- | 1.23 | 1.20, 1.26 | <0.001 |
| Person-level |  |  |  |  |  |  |  |  |  |
| Student | 1.21 | 0.85, 1.72 | 0.296 | 1.20 | 0.74, 1.93 | 0.455 | 1.53 | 1.21, 1.94 | <0.001 |
| Prop. Planned Days | 0.69 | 0.31, 1.55 | 0.362 | 0.86 | 0.29, 2.55 | 0.786 | 0.82 | 0.47, 1.41 | 0.461 |
| Proportion MD | 1.50 | 0.82, 2.76 | 0.185 | 1.06 | 0.48, 2.36 | 0.889 | 1.32 | 0.90, 2.00 | 0.158 |
| *Note.* In all models, HID is the referent event (coded 2). MD= Manner of Drinking protective behavioral strategy; HID=high intensity drinking (i.e., 8+ for females and 10+ for males); HED=heavy episodic drinking (i.e., 4+ for females and 5+ drinks for males); OR=odds ratio; CI=confidence interval, ERR=event rate ratio. Weekend coded 1 or Friday/Saturday and 0 for all other days. Student coded 1 for current 4-year college attendance and 0 for all other. Random effects in the model predicting day type were non-significant and fixed. In the prediction of consequences, random effects that were significant (Study Day, Total Drinks, Any MD, Planned x MD) were retained. The effect of planned drinking is specific to days when one was at their own average proportion of days using MD; *Effect of interest; p-values adjusted following Benjamini-Hochberg procedure. | | | | | | | | | |

| **Supplementary Table 6** |  | | |  | | |  | | |
| --- | --- | --- | --- | --- | --- | --- | --- | --- | --- |
| *Testing Any Serious Harm Reduction (SHR) Strategy as a Moderator of Effects of Planned Drinking on Odds of High-Intensity Drinking* *and Negative Consequences* | | | | | | | | | |
|  | HED vs HID | | | Moderate vs HID | | | # Negative Consequence | | |
|  | OR | 95%CI | *p* | OR | 95%CI | *p* | ERR | 95%CI | *p* |
| Intercept | 3.73 | 2.45, 5.69 | <0.001 | 8.03 | 4.97, 12.98 | <0.001 | 0.41 | 0.32, 0.53 | <0.001 |
| Day-level |  |  |  |  |  |  |  |  |  |
| Any SHR | 0.94 | 0.57, 1.55 | 0.800 | 0.54 | 0.31, 0.92 | 0.024 | 0.86 | 0.66, 1.12 | 0.259 |
| Planned | 0.52 | 0.39, 0.69 | <0.001 | 0.14 | 0.10, 1.20 | <0.001 | 1.39 | 1.19, 1.64 | <0.001 |
| Planned x SHR* | 1.05 | 0.58, 1.89 | 0.871 | 0.86 | 0.42, 1.77 | 0.682 | 1.11 | 0.80, 1.54 | 0.628 |
| Study Day | 0.99 | 0.97, 1.01 | 0.194 | 0.99 | 0.97, 1.00 | 0.104 | 0.98 | 0.98, 0.99 | <0.001 |
| Weekend | 0.53 | 0.41, 0.67 | <0.001 | 0.27 | 0.20, 0.35 | <0.001 | 1.12 | 0.98, 1.27 | 0.095 |
| Total Drinks | --- | --- | --- | --- | --- | --- | 1.23 | 1.20, 1.26 | <0.001 |
| Person-level |  |  |  |  |  |  |  |  |  |
| Student | 1.21 | 0.85, 1.72 | 0.292 | 1.17 | 0.73, 1.89 | 0.509 | 1.60 | 1.26, 2.02 | <0.001 |
| Prop. Planned Days | 0.67 | 0.30, 1.52 | 0.336 | 0.85 | 0.29, 2.51 | 0.771 | 0.71 | 0.42, 1.19 | 0.192 |
| Proportion SHR | 1.17 | 0.67, 2.04 | 0.588 | 0.82 | 0.39, 1.74 | 0.605 | 1.39 | 0.96, 2.02 | 0.082 |
| *Note.* In all models, HID is the referent event (coded 2). SHR= Serious Harm Reduction protective behavioral strategy; HID=high intensity drinking (i.e., 8+ for females and 10+ for males); HED=heavy episodic drinking (i.e., 4+ for females and 5+ drinks for males); OR=odds ratio; CI=confidence interval, ERR=event rate ratio. Weekend coded 1 or Friday/Saturday and 0 for all other days. Student coded 1 for current 4-year college attendance and 0 for all other. Random effects in the model predicting day type were non-significant and fixed. In the prediction of consequences, random effects that were significant (Any SHR, Study Day, Weekend, Total Drinks) were retained. The effect of planned drinking is specific to days when one was at their own average proportion of days using SHR; *Effect of interest; p-values adjusted following Benjamini-Hochberg procedure. | | | | | | | | | |
